# LINC01089 suppresses lung adenocarcinoma cell proliferation and migration via miR-301b-3p/STARD13 axis

**DOI:** 10.1186/s12890-021-01568-6

**Published:** 2021-07-19

**Authors:** Ye Qian, Yan Zhang, Haoming Ji, Yucheng Shen, Liangfeng Zheng, Shouliang Cheng, Xiaomin Lu

**Affiliations:** Department of Oncology, Haian People’s Hospital of Jiangsu Province, No.17 Zhongba Middle Road, Haian, 226600 Jiangsu China

**Keywords:** LINC01089, miR-301b-3p, STARD13, LUAD

## Abstract

**Background:**

Lung adenocarcinoma (LUAD) is one of the most common cancers with high morbidity and mortality worldwide. Long non-coding RNAs (lncRNAs) serve as tumor promoters or suppressors in the development of various human malignancies, including LUAD. Although long intergenic non-protein coding RNA 1089 (LINC01089) suppresses the progression of breast cancer, its mechanism in LUAD requires further exploration. Thus, we aimed to investigate the underlying function and mechanism of LINC01089 in LUAD.

**Methods:**

The expression of LINC01089 in LUAD and normal cell lines was detected. Functional assays were applied to measure cell proliferation, apoptosis and migration. Besides, mechanism experiments were employed for assessing the interplay among LINC01089, miR-301b-3p and StAR related lipid transfer domain containing 13 (STARD13). Data achieved in this study was statistically analyzed with Student’s *t* test or one-way analysis of variance.

**Results:**

LINC01089 expression was significantly down-regulated in LUAD tissues and cells and its overexpression could reduce cell proliferation and migration. Moreover, LINC01089 could regulate STARD13 expression through competitively binding to miR-301b-3p in LUAD. Additionally, rescue assays uncovered that STARD13 depletion or miR-301b-3p overexpression could countervail the restraining effect of LINC01089 knockdown on the phenotypes of LUAD cells.

**Conclusion:**

LINC01089 served as a tumor-inhibitor in LUAD by targeting miR-301b-3p/STARD13 axis, providing an innovative insight into LUAD therapies.

*Trial registration* Not applicable.

**Supplementary Information:**

The online version contains supplementary material available at 10.1186/s12890-021-01568-6.

## Background

Lung cancer, as one of the most common human malignancies, has been identified as the main cause of cancer-associated death in China and even across the world [1, 2]. According to the categorization of lung cancer, it can be divided into small cell lung cancer (SCLC) and non-small cell lung cancer (NSCLC) on the basis of differentiated stages and morphological features [3]. LUAD, which has been identified as the most dominant histological subclass of NSCLC, has high mortality as well as metastasis rate [4–6]. In the past few years, great improvements have been made in the clinical treatment of LUAD, including anti-PD-1/PD-L1 therapy and molecule-targeted therapies [7, 8]. Previous investigations have indicated that the initiation and progression of LUAD involves intricate biological processes that include plentiful genetic and epigenetic alterations [9, 10]. Although multiple molecular genetic researches have been applied in LUAD, the specific molecular mechanisms concerning the progression of LUAD still needs to be further elaborated.

Long non-coding RNAs (lncRNAs), which are identified as a kind of transcripts with exceeding 200 nucleotides in length, have no capability of coding proteins [11]. Aberrant expression of lncRNAs has been detected in a variety of human cancers, in which ovarian cancer [12], colorectal cancer [13] and LUAD [14] are included. More importantly, dysregulation of lncRNAs has been manifested to be closely associated with the tumorigenesis and development of assorted human tumors. For example, MALAT1 knockdown facilitates the metastatic ability of cells in human breast cancer [15]. Up-regulation of LOXL1-AS1 promotes cell proliferation and cell cycle by targeting miR-541-3p and CCND1 in prostate cancer [16]. LINC01089 is a recently researched lncRNA and it has been uncovered to exert anti-tumor function in breast cancer [17]. However, the possible regulatory mechanism and detailed function of LINC01089 in LUAD still need to be explored.

Through designing and conducting this study, we aimed to investigate the underlying regulatory role of LINC01089 in LUAD by implementing a series of functional assays and mechanism assays, which might provide a meaningful reference for LUAD treatment.

## Methods

### Cell lines and plasmid transfection

LUAD cell lines (PC9, H2073, H-1975, A549; ATCC, Manassas, VA, USA) and human bronchial epithelial cell line (BEAS-2B; ATCC) were cultivated in RPMI 1640 medium (Gibco, Grand Island, NY, USA) at 37 °C with 5% CO_2_. The penicillin, streptomycin and 10% fetal bovine serum (FBS; Gibco) were used as the medium supplements. PC9 cell line was purchased from Mingzhoubio (Ningbo, Zhejiang, China), and other cell lines were all bought from ATCC. The catalogue numbers of the cell lines are listed as follows: PC9 (MZ-0668); H2073 (CRL-5918); H-1975 (CRL-5908); A549 (CCL-185); BEAS-2B (CRL-9609).

For transfection, A549 and H-1975 cells at 80–90% confluence were seeded into 6-well plates and transfected for 48 h by using Lipofectamine 3000 kit (Invitrogen, Carlsbad, CA, USA). Cells stably transfection were screened by utilizing G418 and then applied in subsequent experiments. The pcDNA3.1/LINC01089, pcDNA3.1/STARD13 and control (pcDNA3.1), STARD13-specific shRNAs (sh-STARD13#1/2) and control (sh-NC), together with miR-301b-3p mimics/inhibitor and control (NC mimics/inhibitor), were all procured from RiboBio (Guangzhou, China). In addition, the primary ADC cells were used for in vivo assays with HLC as a control in the study. The sequences were listed as follows:

- sh-NC: 5′-CCGGTTCTTTAAAAAAAAAATTTGTCTCGAGACAAATTTTTTTTTTAAAGAATTTTTG-3′,
- sh-STARD13#1: 5′-CCGGGAGGGAAAAGGTCATCTTTCTCTCGAGAGAAAGATGACCTTTTCCCTCTTTTTG-3′,
- sh-STARD13#2: 5′-CCGGCAGATTCATTAAGAGATGTTACTCGAGTAACATCTCTTAATGAATCTGTTTTTG-3′;
- NC inhibitor: 5′-GCTTTGACAATATCATTTTTTTG-3′,
- miR-301b-3p inhibitor: 5′-GCTTTGACAATATCATTGCACTG-3′;
- NC mimics: 5′-GAGAAAGCAGUUCCUACGAUAUU-3′,
- miR-301b-3p mimics: 5′-CAGUGCAAUGAUAUUGUCAAAGC-3′.

### Real-time RT-qPCR (RT-qPCR)

In line with the manual of Trizol (Invitrogen), total RNA from A549 and H-1975 cells was obtained, centrifuged and washed. After using the Prime Script™ RT Master Mix (TaKaRa, Otsu, Japan), the synthesized cDNA was subjected to SYBR Green I fluorescent method (TaKaRa) on Applied Biosystems 7900 Real‐Time PCR System (Applied Biosystems, Foster City, CA, USA). The relative quantification of samples was tested by the equation 2^−ΔΔCt^. GAPDH or U6 was used as the normalized control. The primer sequences were shown in Table 1.

**Table 1:** The sequences of primers used in RT-qPCR were presented

Genes	Sequences of primers
LINC01089	Forward: 5′-GTGGAAGGAGCAGAACGTGA-3′
	Reverse: 5′-CTTACTTACCCGCTCAGCCC-3′
STARD13	Forward: 5′-CGAGGAGACAGAAATGGGTCA-3′
	Reverse: 5′-TCCACTGCTTTCGCTGTGAAT-3′
miR-301b-3p	Forward: 5′-CAGTGCAATGATATTGTCAAAGC-3′
	Reverse: 5′-CTCTACAGCTATATTGCCAGCCAC-3′
miR-454-3p	Forward: 5′-TAGTGCAATATTGCTTATAGGGTGC-3′
	Reverse: 5′-CTCTACAGCTATATTGCCAGCCAC-3′
miR-301a-3p	Forward: 5′-CAGTGCAATAGTATTGTCAAAGCG-3′
	Reverse: 5′-CTCTACAGCTATATTGCCAGCCAC-3′
miR-130b-3p	Forward: 5′-CAGTGCAATGATGAAAGGGC-3′
	Reverse: 5′-CTCTACAGCTATATTGCCAGCCAC-3′
miR-130a-3p	Forward: 5′-CAGTGCAATGTTAAAAGGGCAT-3′
	Reverse: 5′-CTCTACAGCTATATTGCCAGCCAC-3′
miR-3666	Forward: 5′-CAGTGCAAGTGTAGATGCCG-3′
	Reverse: 5′-CTCTACAGCTATATTGCCAGCCAC-3′
miR-4295	Forward: 5′-CAGTGCAATGTTTTCCTTGGA-3′
	Reverse: 5′-CTCTACAGCTATATTGCCAGCCAC-3′
miR-148b-3p	Forward: 5′-TCAGTGCATCACAGAACTTTGTG-3′
	Reverse: 5′-CTCTACAGCTATATTGCCAGCCAC-3′
miR-152-3p	Forward: 5′-TCAGTGCATGACAGAACTTGG-3′
	Reverse: 5′-CTCTACAGCTATATTGCCAGCCAC-3′
miR-148a-3p	Forward: 5′-TCAGTGCACTACAGAACTTTGTCC-3′
	Reverse: 5′-CTCTACAGCTATATTGCCAGCCAC-3′
miR-27b-3p	Forward: 5′-TTCACAGTGGCTAAGTTCTGCC-3′
	Reverse: 5′-CTCTACAGCTATATTGCCAGCCAC-3′
miR-27a-3p	Forward: 5′-TTCACAGTGGCTAGTTCCGC-3′
	Reverse: 5′-CTCTACAGCTATATTGCCAGCCAC-3′
miR-370-5p	Forward: 5′-CAGGTCACGTCTCTGCAGTTAC-3′
	Reverse: 5′-CTCTACAGCTATATTGCCAGCCAC-3′
miR-665	Forward: 5′-ACCAGGAGGCTGAGCCC-3′
	Reverse: 5′-CTCTACAGCTATATTGCCAGCCAC-3′
GAPDH	Forward: 5′-ACAACTTTGGTATCGTGGAAGG-3′
	Reverse: 5′-GCCATCACGCCACAGTTTC-3′
U6	Forward: 5′-ACGACAAACCTGCTGGTAGC-3′
	Reverse: 5′-TCTGGACGAAGAGGATTCGC-3′

### Cell counting kit-8 (CCK-8) assay

10 μl of CCK-8 reagents (Dojindo Molecular Technologies, Tokyo, Japan) was added to the medium containing A549 and H-1975 cells for 2 h. The microplate reader (Bio-Tek, Winooski, VT, USA) was applied for monitoring the absorbance at wavelength of 450 nm.

### EdU incorporation assay

Cell proliferation of A549 and H-1975 was analyzed via EdU incorporation assay kit (Ribobio). LUAD cells were placed in 96-well plates with 100 μl of 50 μM EdU for 3 h, and then treated with 4% paraformaldehyde and 100 μl of 0.5% Troxin X-100 (×100; Sigma-Aldrich, Miamisburg, OH, USA). Following Apollo® 488 fluorescent staining, nuclei were counterstained with DAPI (Beyotime, Shanghai, China). Thereafter, cells were observed and analyzed with fluorescent microscope (Leica, Wetzlar, Germany).

### Flow cytometry of apoptosis

Cell apoptosis of LUAD cells were measured with the help of FITC Annexin V Apoptosis Kit (BD Biosciences, San Jose, CA, USA). A549 and H-1975 cells treated with trypsin were washed in pre-cooled phosphate buffer saline (PBS). 5 × 10^5^ cells were cultured in 100 μl of 1× binding buffer adding 5 μl of PI and 5 μl of FITC Annexin V at room temperature. After 400 μl of 1× binding buffer was added, Flow Cytometer (BD Biosciences) was utilized to determine cell apoptosis rate.

### Wound healing assay

In the wound healing assay, the collected 5 × 10^5^ A549 or H-1975 cells seeded in 24-well plates were cultivated at 37 °C until cells reached 100% confluence after transfection. Thereafter, cells were scraped by 200 μl sterile micropipette tip, and then cultured at 37 °C for 24 h. After being washed for three times in serum-free medium to clear the detached cells, the scratch was imaged by microscope at the time 0 h and 24 h for analysis.

### Transwell assay for cell migration

This assay was conducted in 24-well Transwell chamber (Corning, Corning, NY, USA) containing 8 μm pore size polycarbonate membrane filter. A549 or H-1975 cells were seeded in the upper chamber with 500 μl of culture medium without FBS, while lower chamber was filled with 500 μl of complete medium. After 24 h of incubation, cells in the lower side were subjected to 4% formaldehyde (Sigma-Aldrich) and 1% crystal violet (Sigma-Aldrich), and then counted under optical microscope (Olympus, Tokyo, Japan) at ×200 magnification.

### Subcellular fractionation assay

The segmentation of nucleus and cytoplasm was performed by PARIS™ kit (Ambion, Austin, TX, USA). 1 × 10^7^ A549 or H-1975 cells were washed on ice and re-suspended in 500 μl pre-cooled cell fractionation buffer for 10 min. The supernatant was reaped as cell cytoplasm after centrifugation, while the nuclear deposit was treated with cell disruption buffer. After collecting nuclear and cytoplasmic fraction, RT-qPCR was performed for quantifying LINC01089, with GADPH and U6 as cytoplasmic and nuclear controls, respectively.

### RNA pull-down assay

Using Pierce Magnetic RNA-Protein Pull-Down Kit (Thermo Fisher Scientific, Waltham, MA, USA), RNA pull-down assay was carried out in A549 and H-1975 cells. Biotinylated LINC01089 probes (LINC01089 biotin probe) were incubated with cell extracts and streptavidin magnetic beads (Invitrogen). The LINC01089 no-biotin probe was used as the control. Finally, the RNA complexes bound to beads were analyzed by RT-qPCR.

### Dual-luciferase reporter analysis

The pmirGLO luciferase vectors (Promega, Madison, WI, USA) containing the firefly reporter gene were formed using the wild-type (WT) or mutant (Mut) LINC01089 sequences with or without miR-301b-3p binding sites, termed as LINC01089-WT/Mut. A549 and H-1975 cells were plated to 24-well plates (5 × 10^4^ cells/well), then co-transfected with LINC01089-WT/Mut for 48 h. Renilla luciferase reporter pRLCMV (Promega) acted as the normalized control. Dual-luciferase Reporter assay system (Promega) was applied to estimate the luciferase activity of each group.

### Western blot

The lysed LUAD cells were separated on 10% SDS-PAGE and transferred electrophoretically onto PVDF membranes (Millipore, Billerica, MA, USA). Following the treatment with 5% non-fat milk (Merck KGaA, Darmstadt, Germany), membranes were cultivated with anti-STARD13 (1:2000 dilution; ab126489; Abcam, Cambridge, MA, USA) or anti-GAPDH (1:2000 dilution; ab128915; Abcam) primary antibodies all night, followed by incubation with HRP-labeled secondary antibody (1:2000 dilution; ab6728; Abcam). Finally, the membranes were exposed to ECL chemiluminescence Detection kit (Millipore). GAPDH was an internal control.

### RNA immunoprecipitation (RIP)

Using Magna RIP RNA Binding Protein Immunoprecipitation Kit (Millipore), RIP assay was carried out in A549 and H-1975 cells using 5 μg anti-AGO2 (03-110; Millipore) or 5 μg anti-IgG antibodies (12-370; Millipore). Anti-IgG group served as a negative control, and cell lysates from RIP lysis buffer were treated with the beads conjugated with above antibodies for 2 h at 4 °C, followed by RNA analysis via RT-qPCR.

### Statistical analysis

All data from experiments including three biological replications were exhibited as the mean ± standard deviation (SD). Data analysis was achieved by Student’s *t*-test (comparison for two groups) while one-way or two-way analysis of variance (ANOVA) applying GraphPad Prism 6.0 (GraphPad, San Diego, CA, USA) was utilized for evaluating the differences among multiple groups. Statistics results with p value below 0.05 were considered to be statistically significant.

## Results

### LINC01089 expression is markedly down-regulated in LUAD cells and its overexpression inhibits LUAD progression

**Fig. 1:**
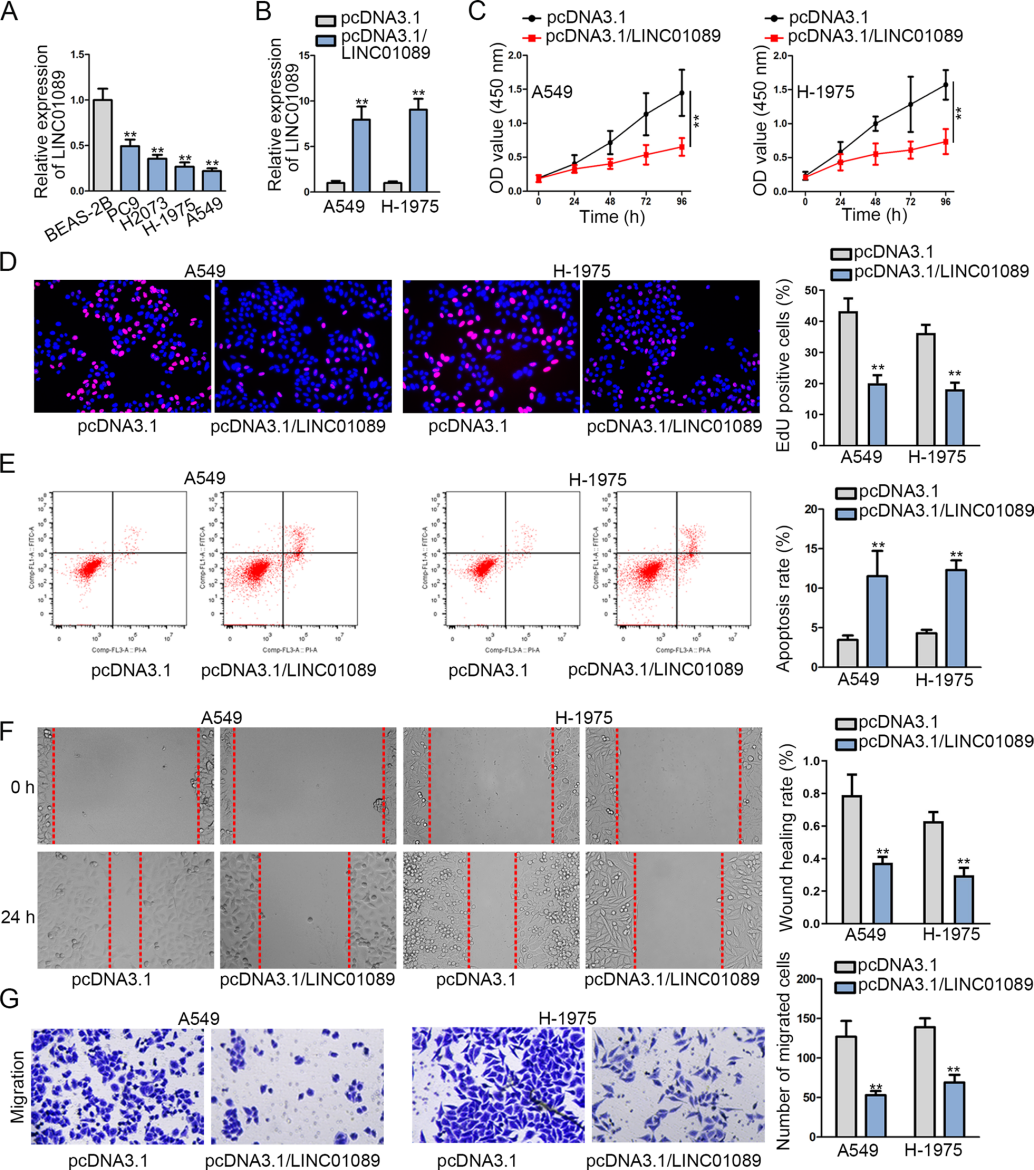
LINC01089 expression is markedly down-regulated in LUAD cells and LINC01089 overexpression suppresses LUAD cell proliferation and migration. **a** RT-qPCR was adopted to detect the LINC01089 expression in LUAD cell lines and BEAS-2B cell. **b** Overexpression efficiency of LINC01089 was detected via RT-qPCR in A549 and H-1975 cells. **c**, **d** Cell proliferation ability of A549 and H-1975 cells was measured through CCK-8 and EdU assays after cells were transfected with pcDNA3.1/LINC01089. **e** The apoptosis ability of pcDNA3.1/LINC01089-transfected cells was tested via flow cytometry analysis. **f**, **g** Transwell and wound healing assays were utilized to detect cell migration capability. The statistical analysis of Fig. 1a was assessed with one-way ANOVA. The statistical analysis of Fig. 1b, d, e, f and g was evaluated with Student’s *t* test. The statistical analysis of Fig. 1C was tested with two-way ANOVA. **P < 0.01

Although LINC01089 has been revealed to be down-regulated in breast cancer cells [17], the expression of it in LUAD cells remains unknown. According to the results shown by GEPIA database (http://gepia.cancer-pku.cn/) and RT-qPCR, LINC01089 was discovered with lower expression in LUAD tissues than that in normal tissues (Additional file 2: Fig.S2A). After that, we observed a significantly down-regulated expression of LINC01089 in LUAD cell lines (PC9, H2073, H-1975 and A549) than that in normal BEAS-2B cell (Fig. 1a). As LINC01089 expression in A549 and H-1975 cells was the lowest, they were kept for follow-up studies. After that, gain-of-function assays were performed to detect the underlying biological function of LINC01089 in LUAD. Before that, RT-qPCR was utilized to detect the overexpression efficiency of LINC01089 in A549 and H-1975 cells (Fig. 1b). Next, CCK-8 assay illustrated that the OD value of cells transfected with pcDNA3.1/LINC01089 was lower when compared with the negative control, suggesting that cell viability of LUAD could be inhibited by LINC01089 overexpression (Fig. 1c). Then, EdU assay revealed that overexpressing LINC01089 led to a decrease in the proliferation of A549 and H-1975 cells since the percentage of EdU positive cells was notably decreased in cells transfected with pcDNA3.1/LINC01089 (Fig. 1d). Additionally, flow cytometry analysis testified that cell apoptosis ability could be enhanced by the overexpression of LINC01089 in A549 and H-1975 cells (Fig. 1e). Moreover, Transwell and wound healing assays uncovered that elevating LINC01089 expression in A549 and H-1975 cells could suppress cell migration (Fig. 1f, g). What’s more, a series of in vivo assays were performed to verify the above functions of LINC01089 in ADC cells. As shown by the results, LINC01089 was found in ADC cells with low expression and the overexpression efficiency of LINC01089 was examined via RT-qPCR (Additional file 1: Fig. S1A-B). After that, functional assays were carried out by us, which demonstrated that LINC01089 overexpression could repress the cell proliferation, migration while enhancing the apoptosis ability of ADC cells (Supplementary Fig. 1C-G). Besides, LINC01089 overexpression could lead to a suppressed tumor growth as well as a decreased tumor weight (Additional file 1: Fig. S1H-J). Taken together, we could confirm that LINC01089 was discovered with low expression in LUAD tissues and cells, and LINC01089 overexpression repressed LUAD cell malignant progression.

### LINC01089 directly binds to miR-301b-3p in LUAD

**Fig. 2:**
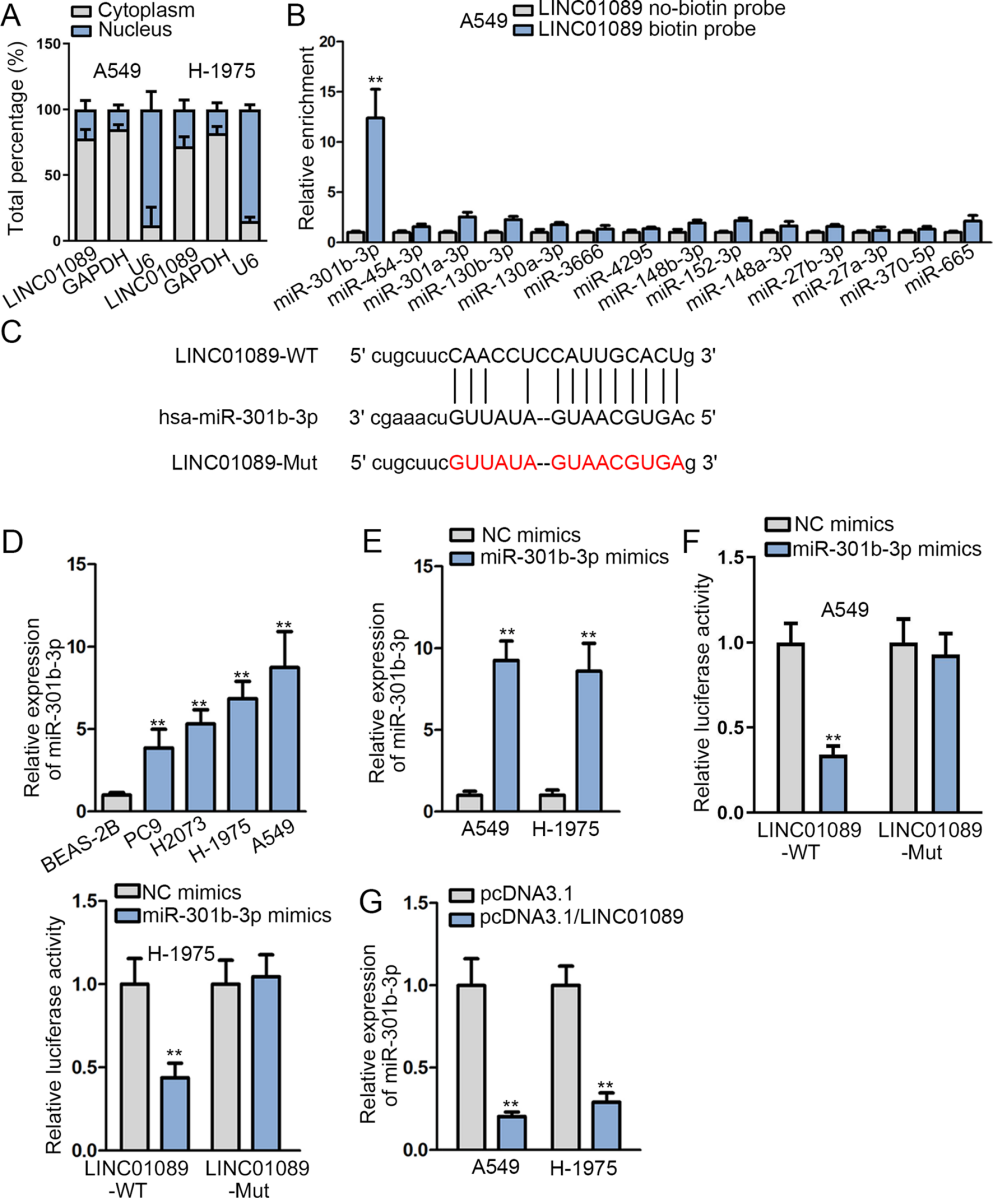
LINC01089 directly binds to miR-301b-3p in LUAD. **a** The subcellular distribution of LINC01089 in A549 and H-1975 cells was detected through subcellular fractionation plus RT-qPCR. **b** The binding capacity between LINC01089 and 14 miRNAs in A549 cells was analyzed through RNA pull-down assay. **c** The binding sites between LINC01089 and miR-301b-3p were predicted via starBase. **d** The expression of miR-301b-3p in LUAD cell lines and BEAS-2B cells was detected. **e** The overexpression efficiency of miR-301b-3p in A549 and H-1975 cells was detected. **f** The interaction between LINC01089 and miR-301b-3p was validated after conducting luciferase reporter assay in A549 and H-1975 cells. **g** The expression of miR-301b-3p was revealed by RT-qPCR after LINC01089 was overexpressed in A549 and H-1975 cells. The statistical analysis of Fig. 2e–g was determined with Student’s *t* test. The statistical analysis of Fig. 2b and d was evaluated with one-way ANOVA. **P < 0.01

In order to explore the potential molecular mechanism of LINC01089 in LUAD, we conducted subcellular fractionation assay to verify the distribution of LINC01089 in LUAD cells. The result demonstrated that LINC01089 was mainly located in the cytoplasm of A549 and H-1975 cells (Fig. 2a). Thus, we speculated that LINC01089 might regulate LUAD progression through sponging miRNA. After searching for the related data from starBase (http://starbase.sysu.edu.cn/index.php), 14 miRNAs were predicted to have a binding relationship with LINC01089 (the detailed information of the 14 miRNAs are listed in Additional file 4: Table S1). After that, RNA pull-down assay revealed an abundant enrichment of miR-301b-3p in LINC01089 biotin probe group, suggesting that miR-301b-3p could bind to LINC01089 in A549 and H-1975 cells (Fig. 2b, Additional file 2: Fig. S2B). Additionally, starBase was applied to predict the binding sites between LINC01089 and miR-301b-3p (Fig. 2c). To investigate how LINC01089 interacted with miR-301b-3p, the expression of miR-301b-3p in LUAD cell lines and BEAS-2B cell and the overexpression efficiency of miR-301b-3p in A549 and H-1975 cells were examined at first (Fig. 2d, e). From our observation, miR-301b-3p overexpression caused a decrease in the luciferase activity of LINC01089-WT whereas no obvious change was observed in the luciferase activity of LINC01089-Mut (Fig. 2f). Furthermore, the expression of miR-301b-3p was notably decreased in A549 and H-1975 cells after the transfection with pcDNA3.1/LINC01089 (Fig. 2g). Taken together, miR-301b-3p directly bound to LINC01089 in LUAD cells.

### LINC01089 regulates STARD13 expression via competitively binding to miR-301b-3p in LUAD

**Fig. 3:**
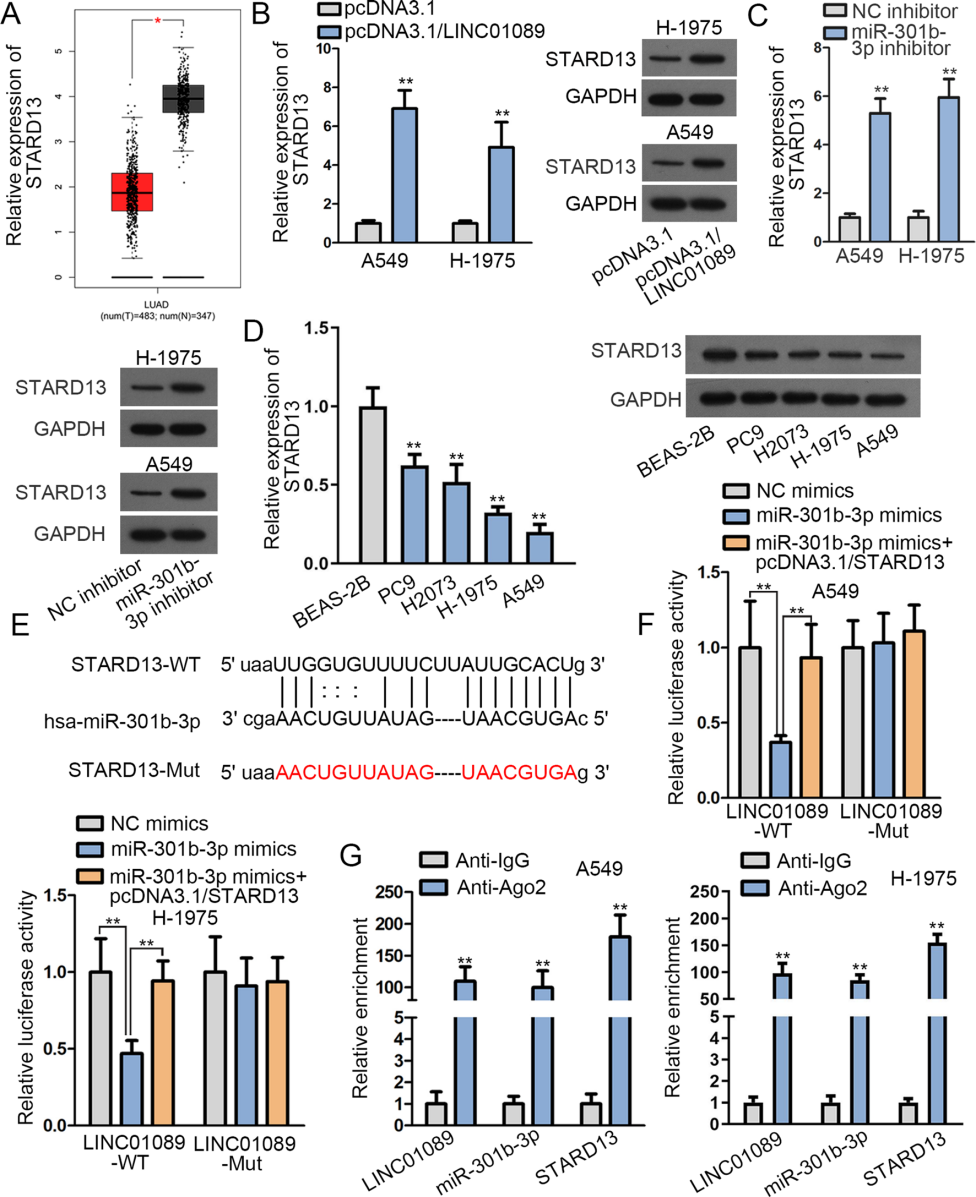
LINC01089 facilitated STARD13 expression via competitively binding with miR-301b-3p in LUAD. **a** In contrast to normal tissues, low expression of STARD13 in LUAD tissues was uncovered via GEPIA database. **b** A significant rise of STARD13 expression in pcDNA3.1/LINC01089-transfected cells was unveiled through RT-qPCR together with western blot analyses. **c** STARD13 expression was revealed through RT-qPCR and western blot analyses after A549 and H-1975 cells were transfected with miR-301b-3p inhibitor. **d** RT-qPCR and western blot analyses validated the expression of STARD13 in LUAD cells and BEAS-2B cell. **e** StarBase was applied to predict the binding sites between STARD13 and miR-301b-3p. **f**, **g** The interaction among LINC01089, miR-301b-3p and STARD13 was verified by luciferase reporter and RIP assays. The statistical analysis of Fig. 3b and c was estimated with Student’s *t* test. The statistical analysis of Fig. 3d, f and g was determined with one-way ANOVA. *P < 0.05, **P < 0.01

To further probe the competing endogenous RNA (ceRNA) mechanism of LINC01089, miRWalk database (http://mirwalk.umm.uni-heidelberg.de/) was consulted and seven mRNAs that might bind to miR-301b-3p was presented (Additional file 5: Table S2). Only STARD13 was uncovered in LUAD tissues with a significantly low expression compared with that in normal tissues (Fig. 3a, Additional file 2: Figure S2C-H). Hence, we selected STARD13 for following studies. After that, we found that the overexpression of LINC01089 resulted in a conspicuous up-regulation of the mRNA and protein level of STARD13 (Fig. 3b, Additional file 3: Figure S3A). Then, we examined the interference efficiency of miR-301b-3p (Additional file 3: Figure S3B). After miR-301b-3p expression was inhibited in A549 and H-1975 cells, we found that the expression of STARD13 mRNA and its protein level were observably enhanced in A549 and H-1975 cells (Fig. 3c, Additional file 3: Figure S3C). Besides, it was revealed that STARD13 expression in LUAD cell lines was signally reduced in contrast to that in BEAS-2B cell (Fig. 3d, Additional file 3: Figure S3D). In addition, starBase was applied to explore the binding sites between STARD13 3′UTR and miR-301b-3p (Fig. 3e). Next, the overexpression efficiency of STARD13 was examined in A549 and H-1975 cells (Additional file 3: Figure S3E). Besides, luciferase reporter assay demonstrated that STARD13 overexpression could reverse the inhibitory effect caused by overexpressing miR-301b-3p on the luciferase activity of LINC01089-WT. As for the luciferase activity of LINC01089-Mut, no evident changes were noted in different groups (Fig. 3f). Furthermore, an abundant enrichment of LINC01089, miR-301b-3p and STARD13 was captured in anti-AGO2 group, indicating that these RNAs co-existed in RNA-induced silencing complexes (RISCs) (Fig. 3g). Therefore, it was confirmed that LINC01089 could regulate STARD13 expression by sponging miR-301b-3p in LUAD.

### LINC01089 represses LUAD progression via miR-301b-3p/STARD13 axis

**Fig. 4:**
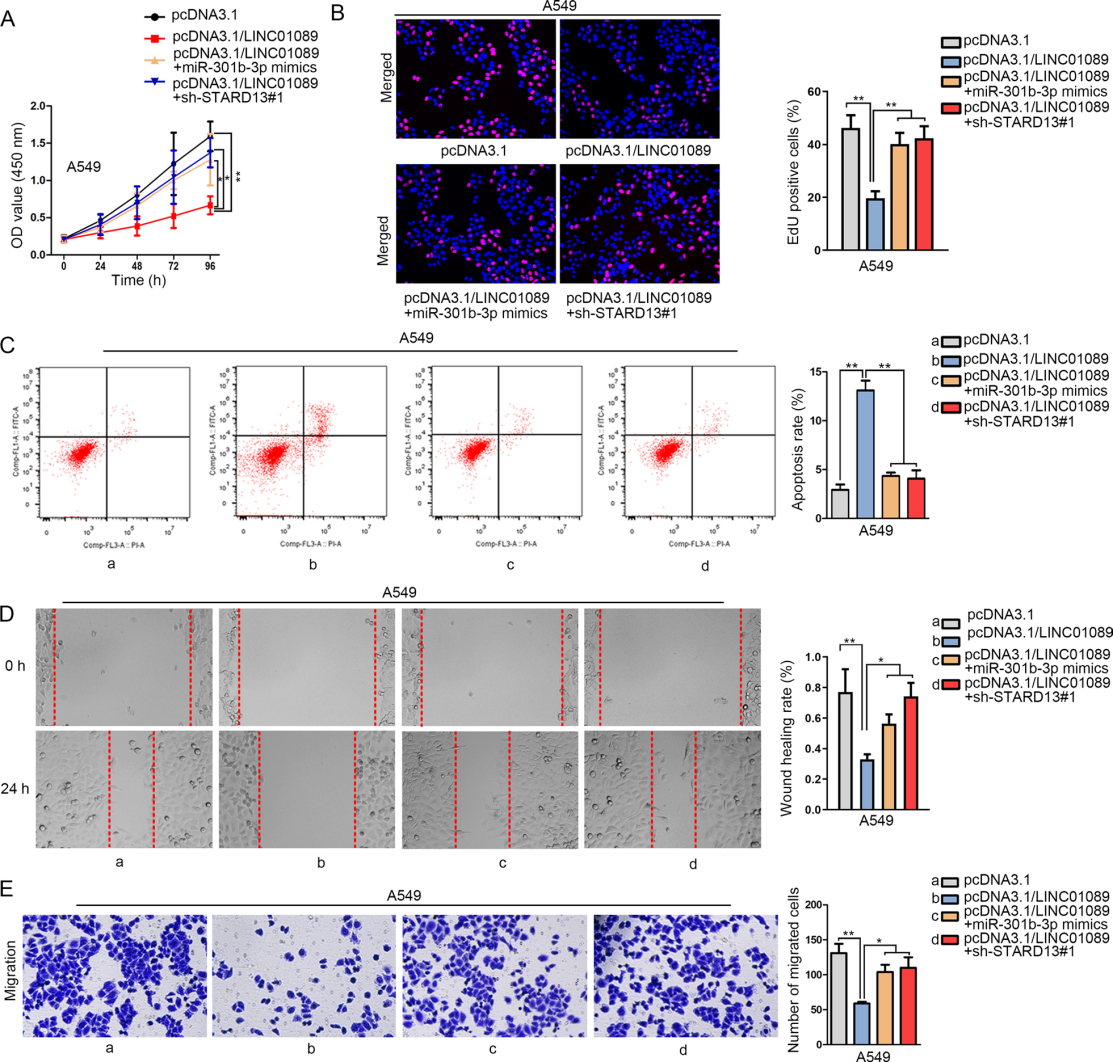
LINC01089 blocked LUAD progression via miR-301b-3p/STARD13 axis. **a**, **b** Co-transfection with sh-STARD13#1 or miR-301b-3p mimics could reverse the restraining effect of pcDNA3.1/LINC01089 on the proliferation ability of A549 cells according to the data from CCK-8 and EdU assays. **c** Silenced STARD13 or up-regulated miR-301b-3p could rescue the facilitating effect of LINC01089 up-regulation on cell apoptosis by flow cytometry analysis. **d**, **e** Co-transfection with sh-STARD13#1 or miR-301b-3p mimics could offset the suppressive effect of pcDNA3.1/LINC01089 on the migratory capability of A549 cells according to the data from Transwell and wound healing assays. The statistical analysis of Fig. 4a was assessed with two-way ANOVA. The statistical significance of Fig. 4b–e was analyzed with one-way ANOVA. *P < 0.05, **P < 0.01

With the intention of validating the existence and role of LINC01089/miR-301b-3p/STARD13 axis in LUAD, we first silenced STARD13 in A549 cells by transfecting the cells with sh-STARD13#1/2 (Additional file 3: Figure S3F). As sh-STARD13#1 exhibited better interference efficiency, it was kept for the following assays. Then data from RT-qPCR analysis confirmed that STARD13 depletion or miR-301b-3p overexpression could countervail the facilitating influence of LINC01089 overexpression on STARD13 expression (Additional file 3: Figure S3G). After that, the weakened cell proliferation ability induced by LINC01089 overexpression was rescued by silencing STARD13 or overexpressing miR-301b-3p (Fig. 4a, b). Besides, STARD13 silencing or miR-301b-3p overexpression could offset the facilitating effect on cell apoptosis caused by LINC01089 up-regulation (Fig. 4c). More importantly, wound healing and Transwell assays verified that STARD13 deficiency or miR-301b-3p overexpression counteracted the repressive influence of LINC01089 overexpression on cell migration (Fig. 4d, e). To sum up, LINC01089/miR-301b-3p/STARD13 axis hindered the progression of LUAD cells.

## Discussion

As the main subtype of NSCLC, LUAD is considered to be among the commonest malignant tumors with high death rate and metastasis rate [4–6]. To improve LUAD therapies, researchers have been dedicated to studying the complicated cellular behaviors of LUAD progression in recent years. Chen et al. has clarified that dysregulation of lncRNAs in lung cancer are critical in regulating the biological processes of this cancer [18]. Existing investigations have further manifested that lncRNAs play pivotal roles in regulating cell proliferation and metastasis in LUAD [19, 20]. In addition, lncRNAs have been revealed to serve as a ceRNA by sponging miRNAs to regulate the expression of protein-coding genes and therefore exerting oncogenic or anti-tumor roles in different kinds of cancers, including LUAD [16, 21]. According to previous studies, LINC01089 has been proved to be a newly confirmed anti-tumor lncRNA in breast cancer [17]. Also, LINC01089 is found to be a lncRNA playing tumor-suppressive role in gastric cancer via regulating miR-27a-3p/TET1 axis [22] and can block the proliferation as well as metastasis of colorectal cancer cells through the regulation of miR-27b-3p/HOXA10 axis [23]. However, the role of LINC01089 in LUAD has not been studied yet. This research first explored the potential regulatory function of LINC01089 in LUAD progression. In this study, LINC01089 was discovered to be down-regulated in LUAD tissues and cells. Furthermore, LINC01089 overexpression repressed LUAD cell proliferation and migration ability while enhancing cell apoptosis.

Existing studies have suggested that lncRNAs may affect LUAD progression via the regulation of certain miRNAs [21, 24]. In this study, owing to the fact that LINC01089 was found mainly in the cytoplasm of LUAD cells, we conjectured that LINC01089 might function as a ceRNA in LUAD by sponging miRNA to regulate the expression of target genes. Multiple reports have clarified that miR-301b-3p exerts the promoting influence on the progression of several human tumors, such as hepatocellular carcinoma [25] and high-grade ovarian serous tumor [26]. Through bioinformatics prediction and molecular mechanism assays, miR-301b-3p was confirmed to bind to LINC01089 in LUAD.

STARD13 has been reported to exert anti-tumor roles in various cancers which include prostate cancer [27] and hepatocellular carcinoma [28]. Besides, Li et al. have revealed the suppressive effect of STARD13-correlated ceRNA network on breast cancer metastasis [29]. In current study, STARD13 was manifested to be directly targeted by miR-301b-3p in LUAD cells. Besides, it was demonstrated that LINC01089 could regulate STARD13 expression by sponging miR-301b-3p in LUAD. In addition, rescue assays revealed that decreased expression of STARD13 or increased expression of miR-301b-3p could offset the restraining effect caused by LINC01089 overexpression on LUAD progression.

## Conclusions

**Fig. 5:**
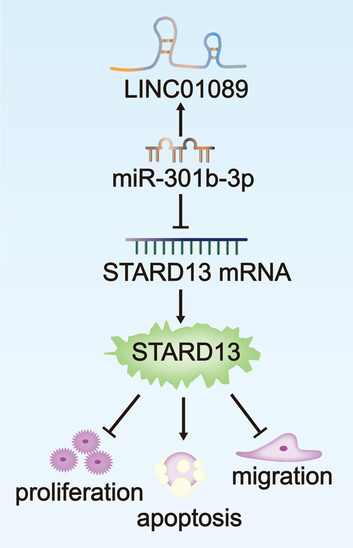
The graphical abstract of our study has been provided for reference

Briefly, all dada obtained in this work suggested that LINC01089 inhibited LUAD progression by targeting miR-301b-3p/STARD13 axis. The finding of LINC01089/miR-301b-3p/STARD13 axis might reveal a novel therapeutic target for the treatment of LUAD. Related graphical abstract has been provided as Fig. 5 for better understanding.

## Supplementary Information

**Additional file 1**. (A) Relative expression of LINC01089 in HLC and ADC cells was examined via RT-qPCR. (B) The overexpression efficiency of LINC01089 was detected through RT-qPCR. (C-G) Functional assays were conducted to examine the proliferation, migration and apoptosis rate of ADC cells after LINC01089 overexpression. (H-J) Tumor growth and weight were measured in ADC cells transfected with pcDNA3.1/ LINC01089. The statistical significance of Supplementary Figure 1A, 1B, 1D-1G and 1J was analyzed with Student’s t test and that of Supplementary Figure 1C and 1H was analyzed with two-way ANOVA. **P < 0.01.

**Additional file 2**. (A) An obvious decrease of LINC01089 expression in LUAD tissues compared with that in normal tissues was obtained from GEPIA database. (B) The binding capacity between LINC01089 and 14 miRNAs in H-1975 cells was analyzed through RNA pull down assay. (C-H) The expression of ARL6IP1, DDX6, CDK19, S1PR2, TP63 and USP32 in LUAD tissues as well as in normal tissues was obtained via GEPIA database. The statistical analysis of Supplementary Figure 2B was estimated with one-way ANOVA. *P < 0.05, **P < 0.01.

**Additional file 3**. (A) Data of western blot assay in Figure 3B were quantified. GAPDH was a normalized control. (B) Interference efficiency of miR-301b-3p was examined via RT-qPCR. (C) Data of western blot assay in Figure 3C were quantified. GAPDH was the normalized control. (D) Data of western blot assay in Figure 3D were quantified. GAPDH was the normalized control. (E) The overexpression efficiency of STARD13 was observed through RT-qPCR. (F) The interference efficiency of STARD13 was detected via RT-qPCR. (G) RT-qPCR was adopted to detect the expression of STARD13 in A549 cell after the co-transfection with sh-STARD13#1 and miR-301b-3p mimics. The statistical significance of Supplementary Figure 3A-C and 3E was analyzed with Student’s t test and that of Supplementary Figure 3D and 3F-G was analyzed with one-way ANOVA. *P < 0.05, **P < 0.01.

**Additional file 4**. According to starBase, 14 miRNAs that could bind to LINC01089 were listed.

**Additional file 5**. After online search of miRWalk database, 7 mRNAs were predicted to be able to bind to miR-301b-3p.

**Additional file 6**. The original protein images of Figure 3B, 3C and 3D.

## Data Availability

The datasets used during the current study are available from the corresponding author on reasonable request. GenBank accession numbers for STARD13, LINC01089, miR-301b-3p and GAPDH are provided as follows: STARD13 (NM_178006.4), LINC01089 (NR_002809.3), miR-301b-3p (NR_030622) and GAPDH (NM_002046.7).

## Abbreviations

lncRNA: Long noncoding RNA
LUAD: Lung adenocarcinoma
SCLC: Small cell lung cancer
NSCLC: Non-small cell lung cancer
LINC01089: Long intergenic non-protein coding RNA 1089
CCK-8: Cell counting kit-8
PBS: Phosphate buffer saline
RIP: RNA immunoprecipitation
ceRNA: Competing endogenous RNA
RISC: RNA-induced silencing complex
STARD13: StAR related lipid transfer domain containing 13
