# Supplementary figures and images for: LINC01089 suppresses lung adenocarcinoma cell proliferation and migration via miR-301b-3p/STARD13 axis
Source: BMC Pulm Med. 2021 Jul 19;21:242. doi: 10.1186/s12890-021-01568-6 (PMC8287768; doi:10.1186/s12890-021-01568-6)

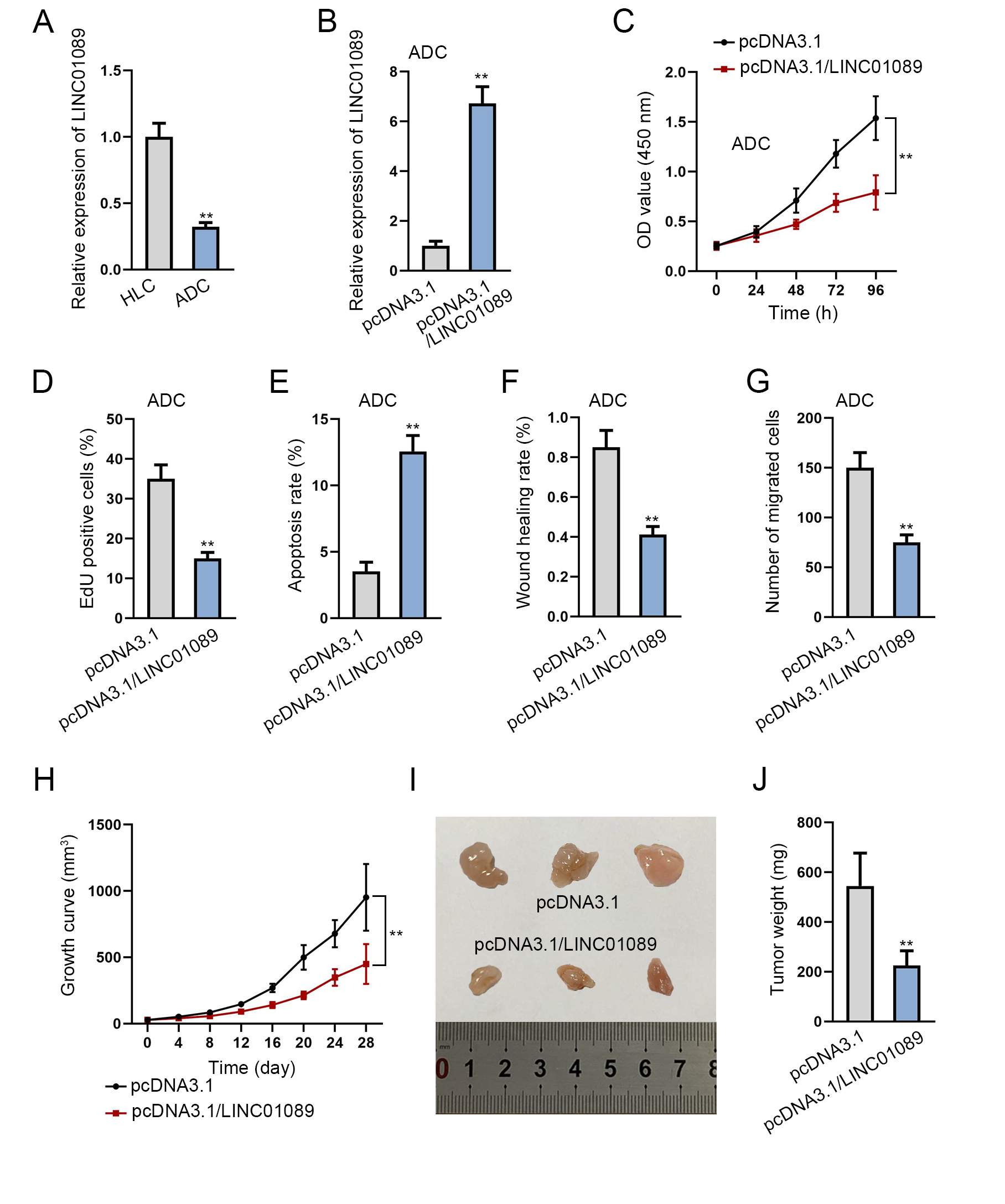

Supplement: Supplementary file 1 — Additional file 1. (A) Relative expression of LINC01089 in HLC and ADC cells was examined via RT-qPCR. (B) The overexpression efficiency of LINC01089 was detected through RT-qPCR. (C-G) Functional assays were conducted to examine the proliferation, migration and apoptosis rate of ADC cells after LINC01089 overexpression. (H-J) Tumor growth and weight were measured in ADC cells transfected with pcDNA3.1/ LINC01089. The statistical significance of Supplementary Figure 1A, 1B, 1D-1G and 1J was analyzed with Student’s t test and that of Supplementary Figure 1C and 1H was analyzed with two-way ANOVA. **P < 0.01. [file 12890_2021_1568_MOESM1_ESM.tif]

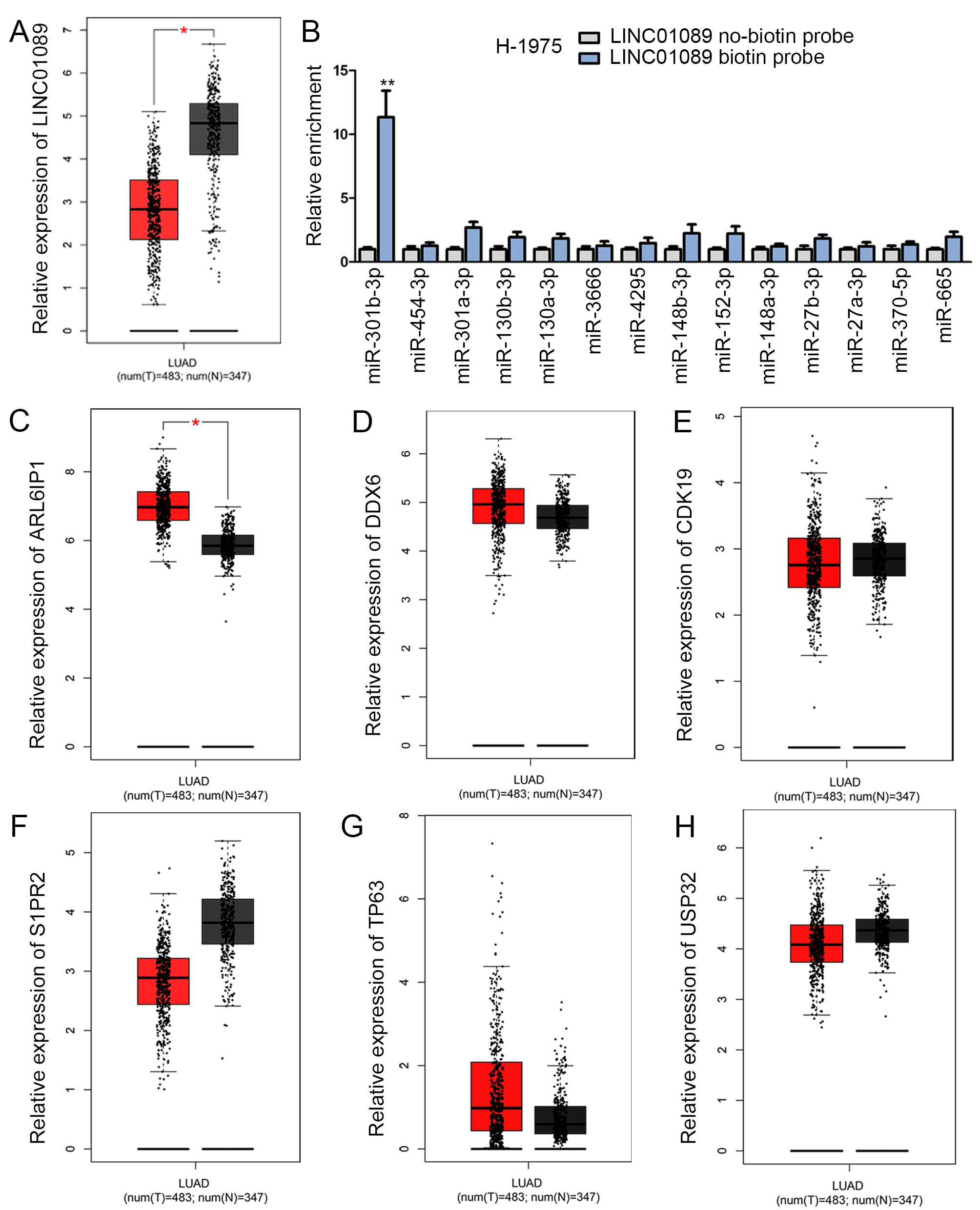

Supplement: Supplementary file 2 — Additional file 2. (A) An obvious decrease of LINC01089 expression in LUAD tissues compared with that in normal tissues was obtained from GEPIA database. (B) The binding capacity between LINC01089 and 14 miRNAs in H-1975 cells was analyzed through RNA pull down assay. (C-H) The expression of ARL6IP1, DDX6, CDK19, S1PR2, TP63 and USP32 in LUAD tissues as well as in normal tissues was obtained via GEPIA database. The statistical analysis of Supplementary Figure 2B was estimated with one-way ANOVA. *P < 0.05, **P < 0.01. [file 12890_2021_1568_MOESM2_ESM.tif]

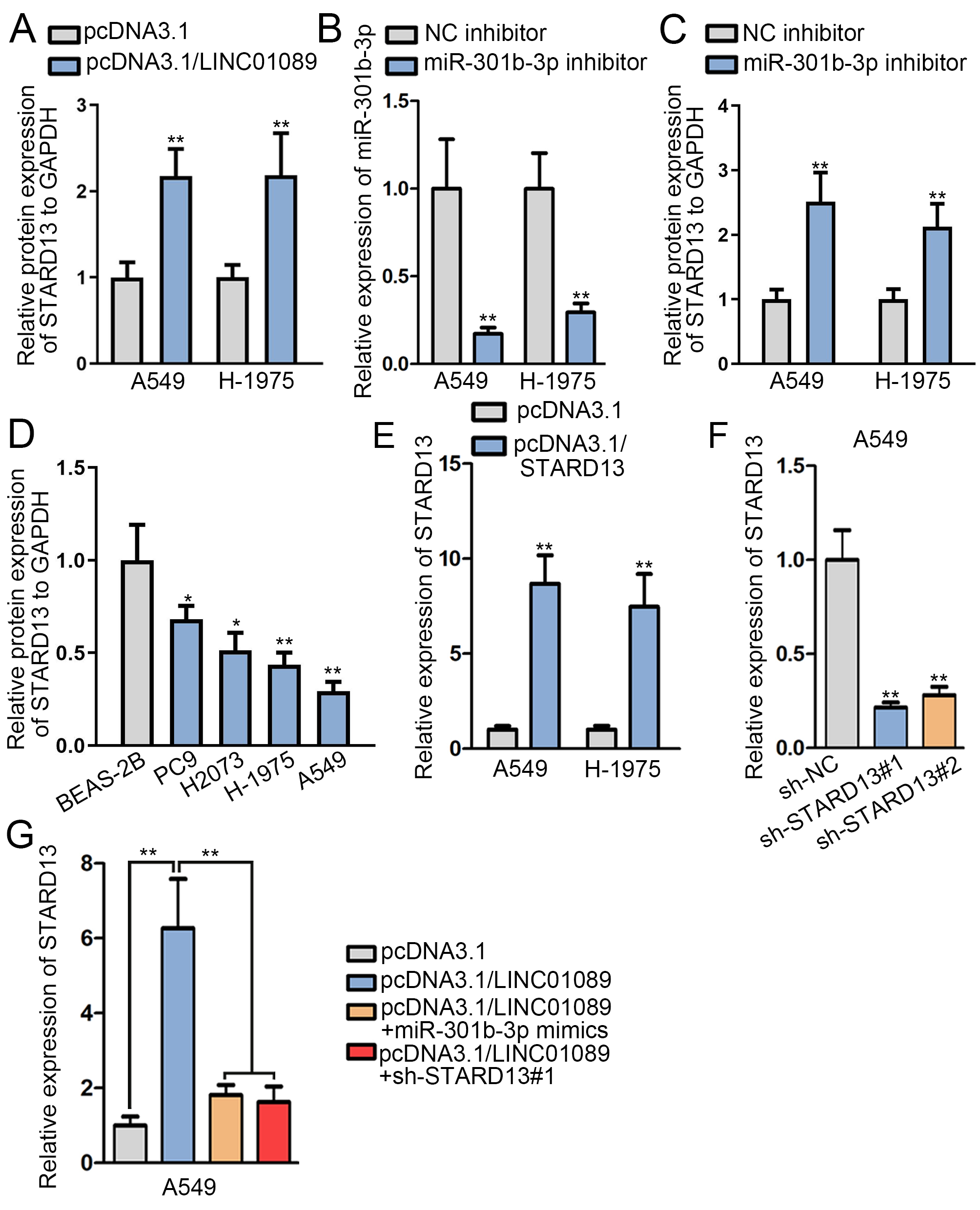

Supplement: Supplementary file 3 — Additional file 3. (A) Data of western blot assay in Figure 3B were quantified. GAPDH was a normalized control. (B) Interference efficiency of miR-301b-3p was examined via RT-qPCR. (C) Data of western blot assay in Figure 3C were quantified. GAPDH was the normalized control. (D) Data of western blot assay in Figure 3D were quantified. GAPDH was the normalized control. (E) The overexpression efficiency of STARD13 was observed through RT-qPCR. (F) The interference efficiency of STARD13 was detected via RT-qPCR. (G) RT-qPCR was adopted to detect the expression of STARD13 in A549 cell after the co-transfection with sh-STARD13#1 and miR-301b-3p mimics. The statistical significance of Supplementary Figure 3A-C and 3E was analyzed with Student’s t test and that of Supplementary Figure 3D and 3F-G was analyzed with one-way ANOVA. *P < 0.05, **P < 0.01. [file 12890_2021_1568_MOESM3_ESM.tif]

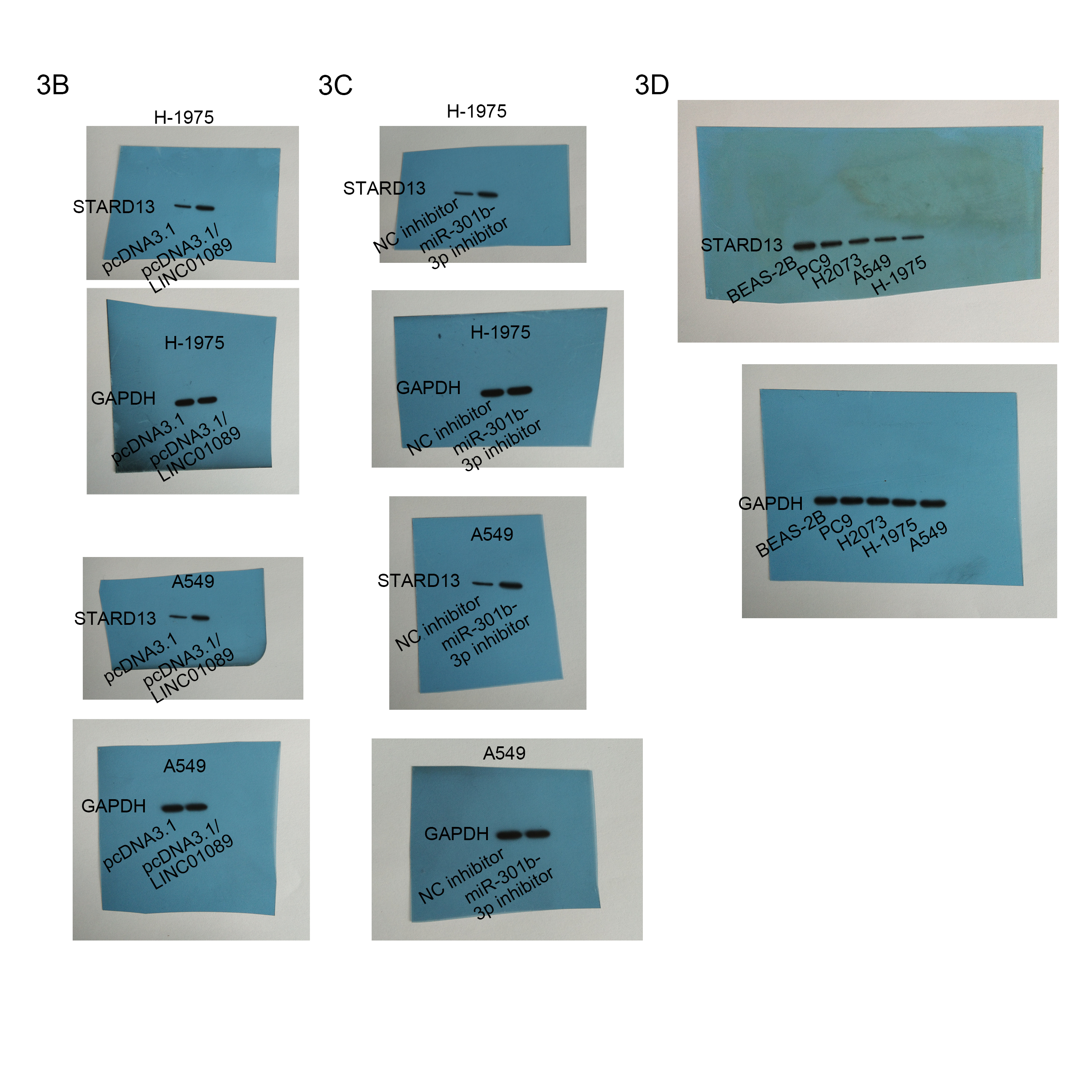

Supplement: Supplementary file 6 — Additional file 6. The original protein images of Figure 3B, 3C and 3D. [file 12890_2021_1568_MOESM6_ESM.tif]
